# On the Effect of Coffee

**Published:** 1853-12

**Authors:** 


### MATERIA MEDICA AND THERAPEUTICS.

*On the Effect of Coffee.* By Dr. ZOBEL.—In a long and interesting article Dr. Zobel discusses the effects of coffee as well as, incidentally, other dietetics. He denies that the use of coffee (and of tea) is to be estimated by the quantity of nitrogen it contains, and shows by calculation how comparatively small a quantity of nitrogen could by this means enter the system. He also denies the accuracy of Rochleder's opinions, that coffee gives rise to the formation of creatine, or if it does so, he questions whether this may not result from its action on the nervous system, and not by immediate transformation of its own substance. With respect to the influence of coffee on the health, he refers to the opinions of a few enthusiasts, such as Jury and Thierry, who have supposed it to be most prejudicial to life. He then inquires what are the chemical changes which occur in the caffein when introduced into the blood. The first change he states to be as follows :

1 equiv. caffein $C_{16}N_4H_{10}O$ makes $\left\{\begin{array}{l}\text{1 equiv. hydrocyanic acid } C_2NH. \\ \text{1 equiv. of a body of the composition } C_{14}N_2H_9O.\end{array}\right.$

The quantity of hydrocyanic acid is very large, and quite enough to appear at first sight a justification of those who have asserted the danger of coffee. But if the examination be continued, this apprehension is dissipated ; another equivalent of caffein acting on the substance formed by the separation of the prussic acid, gives rise to four equivalents of ammonia, the great antidote of the acid, and a third equivalent of caffein and water gives rise to one equivalent of quinine, two of oil of turpentine, and three of urea, while twenty-seven of oxygen remain. The formula is thus given :—

| | | |
|---|---|---|
| 3 equiv. caffein=3($C_{16}N_4H_{10}O_4$) | are | $C_{48}N_{12}H_{30}O_{12}$ |
| 23 equiv. water . . . | are | $H_{23}O_{23}$ |
| | | $C_{48}N_{12}H_{53}O_{35}$ |

Thence are formed—

| | |
|---|---|
| 1 equiv. hydrocyanic acid . . | $C_2N_1H_1$ |
| 4 equiv. ammonia . . . . | $N_4H_{12}$ |
| 1 equiv. quinine . . . . | $C_{20}N_1H_{12}O_2$ |
| 2 equiv. oil of turpentine . . . | $C_{20}\ H_{16}$ |
| 3 equiv. urea . . . . | $C_6N_6H_{12}O_6$ |
| 27 equiv. oxygen . . . . | $O_{27}$ |
| | $C_{48}N_{12}H_{53}N_{35}$ |

By the influence of the ammonia and turpentine, the sedative effects of the hydrocyanic acid are neutralized, and are replaced by a slight stimulant effect on the peripheric nervous system. The ammonia would produce its stimulant effect, and soon pass off, were it not fixed in some measure by the strongest neurine tonic known, the quinine. To these effects of caffein, it is necessary to add those of the tannic acid and volatile oils which the roasted coffee nuts contain, when coffee itself is drank. Dr. Zobel thus confirms the statement of Lehmann, that the amount of urea is increased by the use of caffein. A few pages at the end of the paper are devoted to a consideration of the mode of origin of caffein in the plant, which we omit.—*Brit. & For. Med. Chir. Rev. from Prag. Viertel. Jahrsch.*, 1853.

